# OnabotulinumtoxinA effectiveness on chronic migraine, negative emotional states and sleep quality: a single-center prospective cohort study

**DOI:** 10.1186/s10194-017-0723-4

**Published:** 2017-02-17

**Authors:** Elif Ilgaz Aydinlar, Pinar Yalinay Dikmen, Seda Kosak, Ayse Sagduyu Kocaman

**Affiliations:** Department of Neurology, Acibadem University School of Medicine, Içerenkoy, Kayisdagi Cd, 34752 Atasehir/Istanbul, Turkey

**Keywords:** Chronic migraine, Sleep quality, Headache, Analgesic, MIDAS, DASS-21

## Abstract

**Background:**

OnabotulinumtoxinA (OnabotA) is considered effective in in patients with chronic migraine (CM) who failed on traditional therapies. This study was designed to evaluate the effect of OnabotA injection series on migraine outcome, negative emotional states and sleep quality in patients with CM.

**Methods:**

A total of 190 patients with CM (mean (SD) age: 39.3 (10.2) years; 87.9% were female) were included. Data on Pittsburgh sleep quality index (PSQI), headache frequency and severity, number of analgesics used, Migraine Disability Assessment Scale.

(MIDAS) scores and Depression, Anxiety and Stress Scale (DASS-21) were evaluated at baseline (visit 1) and 4 consecutive follow up visits, each conducted after OnabotA injection series; at week 12 (visit 2), week 24 (visit 3), week 36 (visit 4) and week 48 (visit 5) to evaluate change from baseline to follow up.

**Results:**

From baseline to visit 5, significant decrease was noted in least square (LS) mean headache frequency (from 19.5 to 8.4, *p* = 0.002), headache severity (from 8.1 to 6.1, *p* = 0.017), number of analgesics (from 26.9 to 10.4, *p* = 0.023) and MIDAS scores (from 67.3 to 18.5, *p* < 0.001). No significant change from baseline was noted in global PSOI and DASS-21 scores throughout the study.

**Conclusions:**

Our findings revealed that OnabotA therapy was associated with significant improvement in migraine outcome leading to decrease in headache frequency and severity, number of analgesics used and MIDAS scores. While no significant change was noted in overall sleep quality and prevalence of negative emotional states, patients without negative emotional states at baseline showed improved sleep quality throughout the study.

**Electronic supplementary material:**

The online version of this article (doi:10.1186/s10194-017-0723-4) contains supplementary material, which is available to authorized users.

## Background

Chronic Migraine (CM) is a complex and severely disabling neurological disorder, characterized by occurrence of headache on ≥15 days per month for >3 months with at least five attacks fulfilling criteria of migraine without aura on ≥8 days per month [1].

Having a prevalence of 1–3% documented in population-based studies [2], CM is considered to be a more disabling and burdensome disorder than episodic migraine (EM), as associated with greater migraine-related disability [3], more frequent hospital admissions [4–6], poorer health-related quality of life (HRQoL) [4–7], higher amount of lost work and household productivity [4–6, 8], and increased risk for comorbidities [9] and medication overuse [10].

Increased comorbidity between migraine and several psychiatric conditions has consistently been reported with higher prevalence of mood and anxiety disorders, personality disorders and post-traumatic stress disorder (PTSD) among migraineurs than in the general population [11–13]. Also, increased headache frequency was shown to be correlated with higher rate of depression, anxiety and post-traumatic stress disorder [5, 14–16]. Recent studies also indicate a higher prevalence of poor sleep quality in patients with than without migraine and association of frequency of migraine headache with poor sleep quality [17–22].

This seems notable given the association of co-morbidities with increased burden of CM in terms of productivity loss, impaired HRQoL, healthcare utilization and emotional burden^5^ as well as treatment complications and poor clinical outcomes [13].

Despite availability of preventive and abortive treatments as the mainstays of treatment for migraines, one-third of migraine sufferers remain symptomatic due to frequent partial response to treatment that leads to physical disability and high risk of medication overuse [22–24]. Therefore, development of new and effective therapeutic alternatives is of particular significance in the management of CM [24, 25].

Onabotulinumtoxin A (OnabotA) recently was discovered to be effective in preventing recurrent migraines in patients with CM who failed on traditional therapies [22, 26, 27]. Hence, based on data from PREEMPT (Phase 3 REsearch Evaluating Migraine Prophylaxis Therapy) trials confirming its efficacy in reduction of number of headache days and migraine days in CM patients with favorable safety and tolerability [28–31], OnabotA (155–195 U) is specifically indicated as a prophylactic treatment for CM in adults [26, 32].

The present study was designed to evaluate the effect of OnabotA injection series on migraine outcome, negative emotional states and sleep quality in patients with CM.

## Methods

### Study population

A total of 190 consecutive patients with CM (mean (SD) age: 39.3 (10.2) years; 87.9% were female) were included in this single-center prospective cohort study conducted between May 2012 and May 2016. After baseline visit at study enrollment (visit 1), patients were followed up for 48 weeks via 4 consecutive follow up visits each conducted after a new OnabotA injection series; at week 12 (visit 2), week 24 (visit 3), week 36 (visit 4) and week 48 (visit 5), respectively. Most of the patients had a history of prophylactic treatment for migraine which was either insufficient or was discontinued due to intolerance.

Written informed consent was obtained from each subject following a detailed explanation of the objectives and protocol of the study which was conducted in accordance with the ethical principles stated in the “Declaration of Helsinki” and approved by the Acibadem University Clinical Research Ethics Committee.

### Study parameters

Data on patient demographics (age, gender), educational status, diagnosis (CM, CM + medication overuse), migraine duration (year), family history for migraine, previous migraine treatments, migraine triggers are collected at baseline visit (visit 1). Data on migraine outcome [headache frequency and severity, number of analgesics used and Migraine Disability Assessment Scale (MIDAS) scores], sleep quality [Pittsburgh sleep quality index (PSQI)] and negative emotional states [Depression, Anxiety, and Stress Scale-21 (DASS-21)] were evaluated at baseline and follow up visits. Medication overuse was baseline of simple analgesics on ≥ 15 days. Follow up period was composed of 4 consecutive visits each conducted after a new OnabotA injection series; at week 12 (visit 2), week 24 (visit 3), week 36 (visit 4) and week 48 (visit 5), respectively. Change from baseline to follow up was evaluated based on data from visit 1 to visit 3 (24 weeks) for PSQI, from visit 1 to visit 4 (36 weeks) for DASS-21 scores, while based on data from visit 1 to visit 5 (48 weeks) for headache frequency and severity, number of analgesics used and MIDAS score. Data at visit 2, visit 3, visit 4 and visit 5 refer to changes in parameters observed after 1^st^, 2^nd^, 3^rd^ and 4^th^ OnabotA injection series, respectively.

### Diagnosis of CM

CM was diagnosed based on International Classification of Headache Disorders (third revision) (ICHD-3 beta) diagnostic criteria that require headache occurring on ≥15 days per month for >3 months with at least five attacks fulfilling criteria of migraine without aura on ≥8 days per month [1].

### OnabotA injection series

Administration of OnabotA was performed as 31 fixed-site, fixed-dose intramuscular injections applied at seven specified head and neck muscle points every 12 weeks for a minimum of 24 weeks (2 treatment cycles) according to injection scheme proposed in the PREEMPT studies [28, 29, 33]. We additionally administered OnabotA among occipitalis, temporalis or trapezius muscles using a follow-the-pain strategy.

### MIDAS

The MIDAS is a 5-question tool to quantitatively evaluate the headache-related disability in terms of the number of days in the past 3 months and activity limitations due to migraine. MDAS was developed by Stewart et al.[34] and validated and checked for reliability for Turkish by Ertas et al. [35] The score obtained can be graded as follows: grade I (0 to 5 days) indicative of little or no disability; grade II (6 to 10 days), mild disability; grade III (11 to 20 days), moderate disability; and grade IV (greater than 21 days), severe disability.

### DASS-21

The DASS-21 is a 21-item questionnaire which includes three self-report scales designed to measure the negative emotional states of depression, anxiety and stress. The Depression scale includes items that measure symptoms typically associated with dysphoric mood (e.g., sadness or worthlessness). The Anxiety scale includes items that are primarily related to symptoms of physical arousal, panic attacks, and fear (e.g., trembling or faintness). Finally, the Stress scale includes items that measure symptoms such as tension, irritability, and a tendency to overreact to stressful event. Each item is scored on a 4-point scale (0 = Did not apply to me at all, to 3 = Applied to me very much or most of the time) to rate the extent to which they have experienced each state over the past week. Sum of the score of each item reveals the total score with higher scores indicating greater levels of distress. Each state is categorized into normal/mild/moderate/severe/extremely severe based on cut-off scores recommended for depression (0-4/5-6/7-10/11-13/14+), anxiety (0-3/4-5/6-7/8-9/10+) and stress (0-7/8-9/10-12/13-16/17+) [36–38]. Psychometric properties of the Turkish version of the DASS was studied by Hekimoglu et al. and DASS was shown to be an excellent instrument for measuring features of depression, hyperarousal, and tension in clinical groups [39].

### PSQI

The PSQI, used to assess sleep quality, was developed by Buysse et al. [40] and validated and checked for reliability for Turkish by Agargun et al. [41]. The scale consists of 24 items; eighteen items are scored and yield seven component scores. Each component is scored between 0 and 3 and the total of these scores gives the scale score. The total score ranges between 0 and 21 and the higher the score is, the worse the sleep quality. A total score under 5 indicates ‘good sleep quality’, while a score above 5 shows ‘poor sleep quality’.

### Statistical analysis

Statistical analysis was made using IBM SPSS Statistics (IBM Corp. Released 2012. IBM SPSS Statistics for Windows, Version 21.0, Armonk, NY: IBM Corp). Change over time analysis was based on number of patients with available data on both baseline (visit 1) and follow-up visits (visit 2 to 5), and performed via repeated measures variance analysis and McNemar test for continuous and categorical variables, respectively. Since time to follow up visits showed variability among patients, change over time analysis of continuous variables were adjusted for time (visit 2 = 12 weeks; visit 3 = 24 weeks; visit 4 = 36 weeks; visit 5 = 48 weeks). No specific procedure was defined for missing data. Data were expressed as n(%), mean (standard deviation; SD), median (inter-quartile range, IQR) and mean (lower and upper boundaries of 95% confidence interval; CI) where appropriate. *p* < 0.05 was considered statistically significant.

## Results

### Patient disposition and baseline characteristics

Of 190 patients; 101 (53.2%) attended at least one follow-up visit. Overall 98 (51.6%) patients attended visit 2, 58 (30.5%) patient visit 3, 34 (17.9%) patients visit 4, and 20 (10.5%) patients attend visit 5, which were performed at week 12, week 24, week 36 and week 48, respectively.

During the study period overall four injection series were applied per patient within a total treatment time of 48 weeks (median (IQR) 62.4 (53.8–85.2) weeks).

Most of the participants were university graduates (41.1%), diagnosed with CM per se (48.9%) without triggers (76.8%) and for median (IQR) 3.0 (1.0–10.0) years. Family history for migraine was evident in 28.9% of patients; while most common previous medication was non steroid anti-inflammatory drugs (88.4%). Medication overuse was evident in 76.2% of patients (Table 1).

**Table 1:** Patient characteristics

Age (year), mean (SD)	39.3 (10.2)
Gender, n(%)	
Male	23 (12.1)
Female	167 (87.9)
Total	190 (100.0)
Educational status, n(%)	
Primary school	9 (4.7)
High school	22 (11.6)
University	78 (41.1)
MSc & PhD	9 (4.7)
Missing	72
Diagnosis n(%)	
Chronic migraine	93 (48.9)
Chronic migraine + medication overuse	97 (51.1)
Migraine duration (year), median (IQR)	3.0 (1.0–10.0)
Family history for migraine, n(%)	
Present	55 (28.9)
Absent	61 (32.1)
Missing	74
Previous migraine treatments, n(%)	
Non-steroid anti-inflammatory drugs	168 (88.4)
Antiepileptic	61 (32.1)
Antidepressants	57 (30.0)
Beta blockers	24 (12.6)
Calcium channel blockers	1 (0.5)
Migraine triggers, n(%)	
None	146 (76.8)
At least one trigger	44 (23.2)
Air/weather change	13 (6.8)
Anxiety/depression	15 (7.9)
Fasting	21 (11.1)
Food/beverage	12 (6.3)
Insomnia	33 (17.4)
Menstruation	22 (11.6)
Stress	35 (18.4)

*IQR* interquartile range

### Migraine outcome

Overall, mean (SD; median) headache frequency was 17.9 (7.8;15.0) at visit 1 and 6.8 (5.1;5.0) at visit 5. Median (IQR) headache severity scores were 8.0 (7.0–9.0) and 7.0 (5.0–7.0), number of analgesics used were 20.0 (15.0–30.0) and 5.5 (2.0–10.0) and MIDAS scores were 57.0 (35.5–75.0) and 10.0 (2.0–15.0) at visit 1 and visit 5, respectively (Table 2).

**Table 2:** Overall headache frequency and severity, analgesic use and MIDAS scores

	Headache frequency	Headache severity	Number of analgesics	MIDAS score
	Median (IQR)	Median (IQR)	Median (IQR)	Median (IQR)
Visit 1 (*n* = 185)	15.0(12.0–25.0)	8.0(7.0–9.0)	20.0(15.0–30.0)	57.0(35.5–75.0)
Visit 2 (*n* = 89)	5.0(3.0–10.0)	7.0(5.0–8.0)	5.0(2.0–10.0)	10.5(1.5–23.0)
Visit 3 (*n* = 55)	5.0(2.0–10.0)	6.0(5.0–7.0)	4.0(1.0–10.0)	9.0(3.0–24.0)
Visit 4 (*n* = 31)	4.0(1.0–9.0)	6.0(5.0–8.0)	4.0(1.0–10.0)	6.0(2.0–10.0)
Visit 5 (*n* = 19)	5.0(2.0–10.0)	7.0(5.0–7.0)	5.5(2.0–10.0)	10.0(2.0–15.0)

*IQR* inter-quartile range, *n* patient count without missing data

Mean headache frequency was significantly decreased from LS mean 19.5 at visit 1 to 6.8 at visit 2 (*p* < 0.001), to 7.5 at visit 3 (*p* < 0.001), to 5.4 at visit 4 (*p* < 0.001) and to 8.4 at visit 5 (*p* = 0.002) (Table 3).

**Table 3:** Change in severity, analgesic use, MIDAS scores and headache frequency at follow-up visits

		Headache severity	Number of analgesics	MIDAS score	Headache frequency
Baseline	n	89	80	66	89
	LS mean	8.1	26.9	67.3	19.5
Week 12	n	89	80	66	89
	LS mean (p)	6.2 (<0.001)	7.8 (<0.001)	17.4 (0.001)	6.8 (*p* < 0.001)
Week 24	n	52	50	47	55
	LS mean (p)	5.8 (<0.001)	8.7 (<0.001)	15.3 (<0.001)	7.5 (<0.001)
Week 36	n	31	28	24	30
	LS mean (p)	5.8 (<0.001)	5.1 (<0.001)	9.3 (<0.001)	5.4 (<0.001)
Week 48	n	19	18	17	19
	LS mean (p)	6.1 (0.017)	10.4 (0.023)	18.5 (<0.001)	8.4 (0.002)

*LS mean* least square mean, *n* patient count with valid data at each visit, *P p* value of repeated measures variance analysis with reference to baseline

Mean headache severity was significantly decreased from 8.1 at baseline to 6.2 at visit 2 (*p* < 0.001), to 5.8 at visit 3 and visit 4 (*p* < 0.001 for each) and to 6.1 at visit 5 (*p* = 0.017) (Table 3).

Mean number of analgesics used was significantly decreased from 26.9 at baseline to 7.8 at visit 2 (*p* < 0.001), to 8.7 at visit 3 (*p* < 0.001), to 5.1 at visit 4 (*p* < 0.001) and to 10.4 at visit 5 (*p* = 0.023) (Table 3).

Mean MIDAS score was decreased significantly from 67.3 at baseline to 17.4 at visit 2 (*p* < 0.001), to 15.3 at visit 3 (*p* < 0.001), to 9.3 at visit 4 (*p* < 0.001) and to 18.5 at visit 5 (*p* < 0.001) (Table 3).

### Negative emotional states

DASS-21 revealed normal scores for depression (60.0, 52.0, 60.0 and 57.1%), anxiety (56.5, 51.5, 41.2 and 57.1%) and stress (51.8, 54.5, 29.4 and 42.9%) in similar percentage of patients at visit 1, visit 2, visit 3 and visit 4, respectively (Table 4).

**Table 4:** DASS-21 anxiety, depression and stress scores at study visits

	DASS-21-Depression score, n (%)				DASS-21-Anxiety score, n (%)				DASS-21-Stress score, n (%)			
Severity	Visit 1	Visit 2	Visit 3	Visit 4	Visit 1	Visit 2	Visit 3	Visit 4	Visit 1	Visit 2	Visit 3	Visit 4
Normal	51 (60.0)	13 (52.0)	9 (60.0)	4 (57.1)	48 (56.5)	17 (51.5)	7 (41.2)	4 (57.1)	44 (51.8)	18 (54.5)	5 (29.4)	3 (42.9)
Mild	10 (11.8)	6 (24.0)	2 (13.3)	0 (0)	9 (10.6)	5 (15.2)	3 (17.6)	1 (14.3)	14 (16.5)	6 (18.2)	5 (29.4)	1 (14.3)
Moderate	15 (17.6)	2 (8.0)	3 (20.0)	2 (28.6)	13 (15.3)	5 (15.2)	6 (35.3)	1 (14.3)	16 (18.8)	5 (15.2)	6 (35.3)	2 (28.6)
Severe	6 (7.1)	3 (12.0)	1 (6.7)	0 (0)	10 (11.8)	4 (12.1)	0 (0)	0 (0)	7 (8.2)	4 (12.1)	1 (5.9)	1 (14.3)
Extremely severe	3 (3.5)	1 (4.0)	0 (0)	1 (14.3)	5 (5.9)	2 (6.1)	1 (5.9)	1 (14.3)	4 (4.7)	0 (0)	0 (0)	0 (0)
Total	85 (100)	25 (100)	15 (100)	7 (100)	85 (100)	33 (100)	17 (100)	7 (100)	85 (100)	33 (100)	17 (100)	7 (100)

Mild-to-moderate depression (29.4% at visit 1, 28.6% at visit 4), anxiety (25.9% at visit 1, 28.6% at visit 4) and stress (35.3% at visit 1, 42.9% at visit 4) was evident in remarkable percentage of patients throughout the study period, while severe-to-extremely severe depression (10.6% at visit 1, 14.3% at visit 4), anxiety (17.7% at visit 1, 14.3% at visit 4) and stress (12.9% at visit 1, 14.3% at visit 4) were also noted in less than 15% of patients.

Based on patients with valid data for both baseline and follow up visits, no significant change was noted in percentage of patients categorized to have normal anxiety, depression and stress scores at each follow up visit (Table 5).

**Table 5:** Change in DASS-21 anxiety, depression and stress scores from baseline at study visits

Severity, n (%)	Change at visit 2		Change at visit 3		Change at visit 4	
DASS-21-Depression score	Visit 1	Visit 2	Visit 1	Visit 3	Visit 1	Visit 4
Normal	14 (56.0)	13 (52.0)	8 (53.3)	9 (60.0)	4 (57.1)	4 (57.1)
Abnormal	11 (44.0)	12 (48.0)	7 (46.7)	6 (40.0)	3 (42.9)	3 (42.9)
Total	25 (100)	25 (100)	15 (100)	15 (100)	7 (100)	7 (100)
*P* value^a^	1.000		1.000		1.000	
DASS-21-Anxiety score	Visit 1	Visit 2	Visit 1	Visit 3	Visit 1	Visit 4
Normal	18 (54.5)	17 (51.5)	7 (41.2)	7 (41.2)	3 (42.9)	4 (57.1)
Abnormal	15 (45.5)	16 (48.5)	10 (58.8)	10 (58.8)	4 (57.1)	3 (42.9)
Total	33 (100)	33 (100)	17 (100)	17 (100)	7 (100)	7 (100)
*P* value^a^	1.000		1.000		1.000	
DASS-21-Stress score	Visit 1	Visit 2	Visit 1	Visit 3	Visit 1	Visit 4
Normal	18 (54.5)	18 (54.5)	4 (23.5)	5 (29.4)	3 (42.9)	3 (42.9)
Abnormal	15 (45.5)	15 (45.5)	13 (76.5)	12 (70.6)	4 (57.1)	4 (57.1)
Total	33 (100)	33 (100)	17 (100)	17 (100)	7 (100)	7 (100)
*P* value^a^	1.000		1.000		1.000	

^a^McNemar test Comparisons for visit 5 is not performed due to small number patients with valid data

### Sleep quality

Overall, median (IQR) global score at visit 1 and visit 5 were 9.0 (5.0–12.0) and 4.0 (1.0–7.0), respectively (Table 6).

**Table 6:** Overall PSQI scores at study visits

	Global score	Sleep quality, n(%)		Component scores						
				Subjective sleep quality	Sleep latency	Sleep duration	Habitual sleep efficiency	Sleep disturbances	Use of sleep medications	Daytime dysfunction
	Median (IQR)	Good	Poor	Median (IQR)	Median (IQR)	Median (IQR)	Median (IQR)	Median (IQR)	Median (IQR)	Median (IQR)
Visit 1 (*n* = 59)	9.0 (5.0–12.0)	16 (27.1)	43 (72.9)	2.0 (1.0–2.0)	1.0 (1.0–2.0)	1.0 (0.0–2.0)	1.0 (0.0–2.0)	2.0 (1.0–2.0)	0.0 (0.0–1.0)	1.0 (0.0–2.0)
Visit 2 (*n* = 28)	7.0 (3.0–9.5.0)	11 (39.3)	17 (60.7)	1.0 (1.0–2.0)	1.0 (1.0–2.0)	1.0 (0.0–2.0)	0.5 (0.0–2.0)	1.0 (1.0–2.0)	0.0 (0.0–1.0)	1.0 (0.0–2.0)
Visit 3 (*n* = 14)	10.0 (5.0–15.0)	4 (28.6)	10 (71.4)	2.0 (1.0–2.0)	2.0 (0.0–2.0)	2.0 (1.0–3.0)	1.5 (1.0–2.0)	2.0 (1.0–2.0)	0.0 (0.0–0.0)	2.0 (0.0–2.0)
Visit 4 (*n* = 7)	8.0 (7.0–15.0)	1 (14.3)	6 (85.7)	1.0 (1.0–2.0)	1.0 (0.0–2.0)	2.0 (1.0–3.0)	2.0 (1.0–3.0)	1.0 (1.0–2.0)	0.0 (0.0–2.0)	2.0 (1.0–2.0)
Visit 5 (*n* = 3)	4.0 (1.0–7.0)	2 (66.7)	1 (33.3)	1.0 (0.0–1.0)	0.0 (0.0–1.0)	0.0 (0.0–1.0)	0.0 (0.0–1.0)	1.0 (1.0–2.0)	0.0 (0.0–0.0)	1.0 (0.0–2.0)

*IQR* inter-quartile range (percentile 25 – percentile 75), *SD* standard deviation, *n* patient count without missing data

Based on patients with valid data for both baseline and follow up visits, no significant difference was noted in global scores and thus overall sleep quality from baseline to visit 2 or visit 3. Considering component scores, mean (95% CI lower bound-upper bound) scores for subjective sleep quality (from baseline 1.7 (1.4–2.0) to 1.1 (0.8–1.5) at visit 2, *p* = 0.002), sleep latency (from baseline 1.7 (1.4–2.0) to 1.1 (0.8–1.5 at visit 2, *p* = 0.002) and sleep disturbance (from baseline 1.7 (1.4–2.0) to 1.2 (0.9–1.6) at visit 2, *p* = 0.013) components improved significantly from baseline to visit 2 (Table 7).

**Table 7:** Change in PSQI scores at follow up visits

		Global score	Sleep quality		Component scores						
					Subjective sleep quality	Sleep latency	Sleep duration	Habitual sleep efficiency	Sleep disturbance	Use of sleep medication	Daytime dysfunction
		Mean (95% CI)	n(%)		Mean (95% CI)						
Visit 2 (week 12)	n^a^	25	28		24	24	24	24	24	24	24
	baseline	9.5(7.8;11.3)	good	9 (32.1)	1.7(1.4;2.0)	1.7(1.4;2.0)	1.3(0.8;1.8)	1.4(0.9;2.0)	1.7(1.4;2.0)	0.6(0.1;1.0)	1.4(0.8;1.9)
			poor	19 (67.9)							
	current	8.0(5.8;10.1)	good	11 (39.3)	1.1(0.8;1.5)	1.1(0.8;1.5)	1.3(0.9;1.8)	1.3(0.7;1.9)	1.2(0.9;1.6)	0.7(0.2;1.2)	1.0(0.6;1.4)
			poor	17 (60.7)							
	*p* value	0.185^1^		0.727^2^	**0.002** ^**1**^	**0.002** ^**1**^	0.824^1^	0.724^1^	**0.013** ^**1**^	0.531^1^	0.192^1^
Visit 3 (week 24)	n^a^	13	13		13	13	13	13	13	13	13
	baseline	9.4(6.8;12.1)	good	3 (21.4)	1.8(1.3;2.3)	1.8(1.3;2.3)	1.3(0.5;2.1)	1.6(0.8;2.4)	1.8(1.4;2.2)	0.4(−0.;1.2)	1.3(0.3;2.4)
			poor	11 (78.6)							
	current	10.5(6.0;15.0)	good	4 (28.6)	1.7(1.2;2.2)	1.7(1.2;2.2)	1.6(0.6;2.6)	1.6(0.7;2.4)	1.8(1.1;2.4)	0.9(−0.1;1.8)	1.7(0.8;2.5)
			poor	10 (71.4)							
	*p* value	0.531^1^		1.000^2^	0.590^1^	0.590^1^	0.570^1^	0.940^1^	0.882^1^	0.278^1^	0.205^1^

*CI* confidence interval

^a^number of patients without missing data (data available for both baseline and the specific follow up visit)

^1^Repeated measures variance analysis, ^2^McNemar test. Comparisons for visit 4 and, visit 5 could not be performed due to small number patients with valid data

Values in bold indicate statistical significance (*p* <0.05)

### Sleep quality with respect to DASS-21 scores

Good sleep quality was noted at visit 1, visit 2 and visit 3 34.3, 40.0 and 57.1% of patients with normal depression scores at visit 1, in 38.7, 45.5 and 50.0% of patients with normal anxiety scores at visit 1, and in 33.3, 46.2 and 60.0% of patients with normal stress scores at visit 1, respectively (Table 8).

**Table 8:** Sleep quality with respect to DASS-21 anxiety, depression and stress scores

	Visit 1			Visit 2			Visit 3		
	good SQ	poor SQ	Total	good SQ	poor SQ	Total	good SQ	poor SQ	Total
	n(%)								
DASS 21-depression score									
Normal	12 (34.3)	23 (65.7)	35 (100)	4 (40.0)	6 (60.0)	10 (100)	4 (57.1)	3 (42.9)	7 (100)
Abnormal	4 (16.7)	20 (83.3)	24 (100)	2 (22.2)	7 (77.8)	9 (100)	0 (0)	6 (100)	6 (100)
Total	16 (27.1)	43 (72.9)	59 (100)	6 (31.6)	13 (68.4)	19 (100)	4 (30.8)	9 (69.2)	13 (100)
DASS 21-anxiety score									
Normal	12 (38.7)	19 (61.3)	31 (100)	5 (45.5)	6 (54.5)	11 (100)	3 (50.0)	3 (50.0)	6 (100)
Abnormal	4 (14.3)	24 (85.7)	28 (100)	3 (25.0)	9 (75)	12 (100)	1 (14.3)	6 (85.7)	7 (100)
Total	16 (27.1)	43 (72.9)	59 (100)	8 (34.8)	15 (65.2)	23 (100)	4 (30.8)	9 (69.2)	13 (100)
DASS 21-stress score									
Normal	10 (33.3)	20 (66.7)	30 (100)	6 (46.2)	7 (53.8)	13 (100)	3 (60.0)	2 (40.0)	5 (100)
Abnormal	6 (20.7)	23 (79.3)	29 (100)	2 (20)	8 (80)	10 (100)	1 (12.5)	7 (87.5)	8 (100)
Total	16 (27.1)	43 (72.9)	59 (100)	8 (34.8)	15 (65.2)	23 (100)	4 (30.8)	9 (69.2)	13 (100)

*SQ* sleep quality

Visit 4 to 5 was not evaluated because of small number of data

### Adverse events and treatment alterations under OnabotA therapy

OnabotA therapy was associated with minor and temporary side effects (e.g., asymmetry of the position of the eyebrows and neck ache) in some patients. In one patient who has a very thin cervical region, dysphagia and difficulty in swallowing appeared while regressed after the third week of therapy. Treatment was continued in this patient with omission of further injections to the cervical area.

Treatment alterations included discontinuation of OnabotA therapy (*n* = 2), addition of antiepileptic (*n* = 3) or SSRI (*n* = 9) medications and discontinuation of ongoing antiepileptic (*n* = 3) or SSRI (*n* = 1) medications.

## Discussion

Our findings revealed that administration of four OnabotA injection series over 48 weeks in chronic migraineurs was associated with a significant improvement in all migraine parameters including headache frequency and severity, number of analgesics used and MIDAS scores, while no significant change from baseline was noted in overall sleep quality and prevalence of negative emotional states. Patients without negative emotional states at baseline showed improved sleep quality throughout the study.

Significant reduction in monthly headache frequency, headache severity, number of analgesics used and MIDAS scores in our cohort support the efficacy of OnabotA in reduction of the frequency, intensity, and duration of chronic migraines as well as decreasing medication overuse among CM patients who failed on traditional preventive therapies [22, 25–27, 42–44].

Reduction in MIDAS scores, indicating lesser amount of lost work and personal time due to migraine [6], after OnabotA therapy in our cohort seems notable given that a day lived with severe migraine is considered to be as disabling as a day lived with dementia, quadriplegia or acute psychosis and more disabling than blindness, paraplegia, angina or rheumatoid arthritis [4, 45, 46].

CM has been associated with a high intake of abortive medications with estimated analgesic overuse in 50–80% of patients with CM that may lead to the development of medication overuse headache (MOH) [47]. Given the similar rates of medication overuse (76.2%) in our cohort at baseline, reduction in in number of analgesics used starting from the first injection and consistently throughout the study period emphasizes the potential role of OnabotA therapy in prevention of MOH via headache episode reduction [25, 42, 48–50].

In terms of monthly headache frequency, headache severity, number of analgesics used and MIDAS scores our findings support the association of OnabotA therapy with rapid improvement in migraine parameters usually after the first session as reported in past studies [26, 27, 30, 51–53].

The rapid improvement of migraine parameters after OnabotA therapy has been linked to the combined pharmacological and the placebo effect at the beginning of the treatment [51, 52], while increased benefit offered via repeated OnabotA injections over time is associated with a prophylactic cumulative effect [31, 54]. Also, patients who respond well to therapy early have been suggested to maintain and continue these reductions over at least 2 years [27, 55–57], while limited data are available on long-term efficacy of OnabotA therapy with evaluation of efficacy only up to1 year in most of clinical trials [42, 44, 50].

Alike to our findings, use of four OnabotA injections over 48 weeks in CM patients was reported to be associated with significant improvement in monthly headache days, migraine days and medication days that continued throughout the entire study period [42]. Nonetheless, in a longer-term study with seven OnabotA injections in CM patients, based on a decrease in initial efficacy of OnabotA therapy after the third injection, authors concluded that actual improvement and amelioration of daily headache under OnabotA therapy needs several months to be consolidated [52].

Long-term efficacy of OnabotA in CM patients has recently been evaluated in some studies based on administration of OnabotA therapy for 2 years [25], 4 years [43] and 7–9 injections series [52, 58]. However, while finding are consistent regarding long-term efficacy of therapy with no serious adverse events in responders, they varied in terms of durability of benefit, non-responder rates and the length and necessity of withdrawal of treatment at scheduled times [25, 43, 52, 58].

Both depression and anxiety have been proposed to be a risk factor for migraine chronification [13, 59, 60], while migraine headache frequency and headache severity were reported to be associated with higher rate of depression and anxiety disorders [4, 5, 14, 61]. Accordingly, identification of abnormal scores for depression, anxiety and stress in almost half of patients in our cohort is in line with higher rates of self-reported mood and anxiety disorders in patients with CM as compared with general population and EM patients [4, 5, 13, 14] with more than 40.0% of CM patients to meet criteria for moderate to severe anxiety and depression [4, 13].

Reduction in headache frequency via 24-week OnabotA therapy was reported to be associated with reduction in depression (via Beck Depression Inventory-II) and anxiety scores (Generalized Anxiety Disorder-7 scale) in a past among CM patients [62]. In fact, even a direct effect of OnabotA injection in amelioration of depressive symptoms has been suggested that probably occur via the facial feedback mechanism with consideration of OnabotA as a safe adjunctive treatment to pharmacotherapy for major depressive disorder [63].

In this regard, it seems notable that despite significant reductions obtained via OnabotA therapy in both monthly headache frequency and headache severity in our cohort, no significant change from baseline occurred in depression, anxiety and stress rates throughout the study.

High rates of negative emotional states regardless of the ongoing OnabotA therapy in our patients seems notable given the impact of psychiatric comorbidities on disease prognosis, treatment, and clinical outcomes and higher prevalence of severe headache-related disability, headache impact and poor quality of life in migraineurs with than without psychiatric comorbidities [11, 13, 64]. Hence our findings emphasize consideration of co-morbid psychiatric disorders in diagnostic evaluation and formulation of treatment plan in CM patients, being aware of the likely negative impact of co-morbid psychiatric disorder on treatment outcomes, adherence and quality of life [5, 14, 65].

Identification of poor sleep quality at baseline in 72.9% of our patients seems consistent with high prevalence (30–79%) of co-morbid poor sleep quality among migraineurs [20, 66–68], particularly in those with 8 or more migraine days per month [17]. Higher prevalence of poor sleep quality was also noted in patients with migraine compared to those without migraine [17–21], while high migraine frequency was shown to correlate with poor sleep quality and a higher prevalence of poor sleepers in chronic migraineurs [21].

In a past study on the effects of OnabotA on jaw motor events during sleep in sleep bruxism patients, no significant change from baseline was noted in usual sleep variables such as sleep efficiency, arousal index, sleep stages, or awakenings per hour during follow-up recordings [69].

Similarly, our findings revealed no direct effect of OnabotA injection on overall sleep quality at week 12 and week 24, while significant improvement in subjective sleep quality, sleep latency and sleep disturbance at week 12. This seems notable given the reported association of higher migraine frequency with higher scores for certain domains of PSQI such as “cannot get to sleep within 30 minutes,” “wake up in the middle of the night or early morning,” and “bad dreams” among migraineurs [21].

Sleep-related migraine is considered to be associated with a more severe and disabling clinical course given the increased mean attack severity and monthly use of symptomatic drugs in patients with than without sleep related migraine despite similar monthly headache frequency [70].

Although data on sleep quality are available for only two OnabotA injections in our cohort, given that patients without negative emotional states at baseline showed improved sleep quality throughout the study, maintenance of co-morbid negative emotional states throughout the study seems to be associated with lack of improvement in overall sleep quality, despite significantly decreased migraine frequency.

Nonetheless, it should also be noted that while the presence of negative emotional states such as depression was reported to be reciprocally associated with poor sleep quality [66, 71] and migraine history and comorbid anxiety and depression were shown as predictors of sleep quality [20] in some studies, poor sleep quality was shown to be associated uniquely with migraine itself, regardless of comorbid depression, anxiety or sleep disorders in other studies, particularly among EM patients [17, 44, 72, 73].

Certain limitations to this study should be considered. First, due to observational nature, non-randomized allocation and thereby the likelihood of main selection bias and confounding is possible. Second, although provide data on real-life clinical practice, potential lack of generalizability seems another important limitation due to single-center design of the study. Third, lack of intervention considering timing and number of follow up visits in accordance with the observational nature caused relatively limited follow-up data and the frequency of patient visits to be not uniform with considerable loss to follow up rate challenging analysis of sleep and mood variables. Fourth, analysis of data on negative emotional states and sleep quality were based on self-report rather than objective measures. However, our analysis was based on use of validated questionnaires along with evidence on high concordance between self-report instruments and clinical diagnosis of psychiatric disorders [13]. Lack of follow up data on sleep quality after second OnabotA injection, lack of data on inter-individual variations in headache characteristics with likely impact on therapeutic response as well as lack of data on adverse events are other limitations which otherwise would extend the knowledge achieved in the current study.

## Conclusions

In conclusion, our findings in a cohort of chronic migraineurs revealed that OnabotA injection was associated with significant improvement in migraine outcome leading to decrease in headache frequency and severity, number of analgesics used and MIDAS scores. While no significant change was noted in overall sleep quality and prevalence of negative emotional states with OnabotA injections, patients without negative emotional states at baseline showed improved sleep quality throughout the study. There is a need for future larger-scale long-term longitudinal studies addressing the durability as well as predictors of efficacy of OnabotA in CM patients.

## Additional file

Additional file 1: Data set. (XLSX 23.6 kb)

## Abbreviations

CM: Chronic migraine
DASS-21: Depression, anxiety, and stress scale-21
HRQoL: Health-related quality of life
ICHD: International classification of headache disorders
MIDAS: Migraine disability assessment scale
MOH: Medication overuse headache
OnabotA: OnabotulinumtoxinA
PREEMPT: Phase 3 research evaluating migraine prophylaxis therapy
PSQI: Pittsburgh sleep quality index
PTSD: Post-traumatic stress disorder
